# Moving from risk communication to food information communication and consumer engagement

**DOI:** 10.1038/s41538-018-0031-7

**Published:** 2018-11-30

**Authors:** Patrick G. Wall, Junshi Chen

**Affiliations:** 1 Institute of Food and Health, University College Dublin, Dublin, Ireland; 2 Chinese Centre for Food Safety and Risk Assessment, Chaoyang, China

**Keywords:** Industry, Business and industry, Society, Social sciences

## Abstract

Consumers in most developed countries have greater access to safer food than ever before, yet the issue of consumer perception on the safety of the food supply, the control infrastructure and existing and new process technologies is often not positive. A series of high profile food incidents, which have been ineffectively managed by both the regulators and the industry, and where there has been a failure to be open and transparent, have sensitised a proportion of consumers to scary stories about the food supply. There has been concomitant damage to consumer confidence in (i) the safety of food, (ii) the food industry’s commitment to producing safe food and (iii) the authorities’ ability to oversee the food chain. Threats to consumers’ health and their genuine concerns have to be addressed with effective risk management and the protection of public health has to be paramount. Dealing with incorrect fears and misperceptions of risk has also to be addressed but achieving this is very difficult. The competencies of social scientists are needed to assist in gaining insights into consumer perceptions of risk, consumer behaviour and the determinants of trust. Conventional risk communication will not succeed on its own and more innovative and creative communication strategies are needed to engage with consumers using all available media channels in an open and transparent way. The digital media affords the opportunity to revolutionise engagement with consumers on food safety and nutrition-related issues.

## Introduction

The public health consequences of contaminated food cannot be underestimated and foodborne diseases are an important cause of morbidity and mortality globally. Food safety cannot be taken for granted and when things go wrong people get ill and some will die. There is no shortage of high profile serious outbreaks to keep a proportion of consumers anxious about food safety. An outbreak of *Listeria monocytogenes* in 2017 and 2018 in South Africa, linked to contaminated polony sausage, resulted in over 1000 laboratory confirmed illnesses and over 180 deaths^1^ and an outbreak of *Escherichia coli* O104:H4 which began in Germany in 2011 resulted in over 4000 cases of whom over 900 developed haemolytic uraemic syndrome, and at least 50 died.^2^ The melamine crisis in China which emerged into the public domain in 2008 resulted in more than 290,000 babies falling ill and six deaths.^3^ While developing countries have a bigger problem coping with foodborne diseases, in the so-called developed countries food safety also remains a public health priority. Even in the United States, despite on-going food safety measures, foodborne illness continues to be a substantial health burden and the CDC estimates that each year 48 million people get sick, 128,000 are hospitalised, and 3000 die from foodborne diseases.^4^ Some food scares do not result in reported illnesses but highlight deficiencies in the control infrastructure, e.g. the 2013 EU horse meat scandal, where beef was replaced with horse meat demonstrated that, in the EU, oversight of the meat industry and its traceability system was not as robust as consumers were led to believe.^5^

A global litany of food scares and scandals has exposed weaknesses in many countries’ food safety control systems and authorities have responded with radical reforms to their food safety infrastructure. Many have introduced stronger food legislation with a greater emphasis on independent risk assessment, robust risk management, and open and transparent risk communication. Greater emphasis has been placed on the responsibility of the industry stakeholders, at all stages of the food chain, to produce safe food.^6^ The Food Safety Modernisation Act was enacted in the US in 2011 and the Safe Food for Canadians Act was introduced in 2012 and is being updated in 2018.^7^ The EU introduced radical reforms in response to the BSE crisis as did the Japanese authorities with the new Food Safety Law in 2003.^8–10^ Since the melamine crisis, China has embarked on a continuous process of strengthening, and streamlining, oversight of the food chain with the new Food Safety Law introduced in 2015, and the on-going institutional reform in 2018.^11,12^ In addition, there is continuous improvement in process controls, improved detection methods and better traceability systems which should be making food safer. Also, better surveillance and early warning systems and greater international collaboration now exist.

Despite these changes and the fact that consumers in most developed countries have greater access to affordable safer food, and a greater diversity in food choice than ever before, the issue of consumer perception of the safety of the food supply, the control infrastructure and existing and new process technologies is often not positive.^13–15^ There will always be substandard operators and this coupled with the increasing ability of the chemical analysts to detect contaminants at lower and lower levels and the improving ability to detect food fraud will continue to show weaknesses in the food chain control systems. There are lessons to be learnt from each non-compliance and loophole in the legislation and addressing these will contribute to continuous improvements in risk management approaches to safeguard both the public’s health and the public’s interests. However, the associated adverse publicity often may undermine consumer confidence further.^16^ We live in an era where the media is global and news, particularly, bad news, is rapidly disseminated. Shoddy and unscrupulous operators in the food chain provide ample ammunition for those wishing to generalise from isolated adverse events to the entire food supply and create sensational stories. In addition, many apparently reputable corporations and companies occasionally make mistakes, produce contaminated product and cause outbreaks of illness that shatter consumer confidence further. For example, in 2018 an outbreak of Salmonella mbandaka associated with Kellogg’s honey snack cereal resulted in 136 cases in 36 US states with 34 hospitalisations.^17^ In 2017 a French infant formula company, Lactalis, had to recall product from over 50 countries after 36 infants in France were infected by *Salmonella agona* after consuming the company’s branded infant formulas.^18^ In 2008 an outbreak of *L. monocytogenes* in Canada associated with cooked ham produced by Maple Leaf Foods resulted in 23 deaths. High profile incidents continue to occur.^19^

## Consumer perception of risk

The consumers’ perceptions of risks are often very different from those of the professionals and, at times, there is a complete disconnect between consumers’ views and the true risks associated with a product or a process. Important determinants of consumer risk perception include whether the hazards are considered to be natural or technological (man-made) in origin, whether the hazards have an acute or chronic impact and the level of trust the consumers have in the messages they are receiving.^20^ Often the public believes that food has zero risk which is never the case and therefore, it is a challenge to explain that food is not sterile which means that there is always a degree of risk that the end user has to manage to avoid illness occurring.

Consumer perception is often framed by the information they are exposed to. With the conventional media feeding off the social media and vice versa, often without time for verification, misinformation, and miss-truths can result in gross over-amplification of the risks to the public’s health and undermine consumer confidence in the safety of the food supply and the systems in place to oversee the food chain.^21–24^

In addition to putting the public’s health, and confidence, at risk, badly managed food safety crises can seriously damage, and even destabilise, political, economic, and social systems in a country, or region, as was seen in the 1999 Belgian dioxin crisis and the epidemic of bovine spongiform encephalitis in the UK.^9,27^

## Reliance on Risk Communication to reassure the public

Perhaps initially when risk communicaton was first introduced by the Codex Alimentarius in 1998, as a component of risk analysis, it was primarily to share the result of risk assessments with risk managers and policymakers and communication with the public was secondary. However increasingly, the authorities have relied on risk communication, one of the three components of Risk Analysis as the main approach to interact with the public in the belief that it would allay their fears, provide reassurance and promote confidence.^25^ Risk communication helped after some of the major scares to reduce consumer anxiety and calm some crises but an unjustifiable level of anxiety remains, and is being maintained, amongst subsets of consumers in many jurisdictions. Adverse publicity and consumer unrest regularly influence governments’ agendas and often policies in proportion to the media coverage, rather than the risk to public health, emerge.^26^ Both GMOs and the use of glyphosate have triggered very polarised opinions in the EU and NGOs and lobby groups advocate for stricter regulatory control of the former and a ban on the later. When different scientists hold opposing opinions on the risk of a technology or a product to public health, this can undermine consumer confidence in the entire food safety infrastructure.^9,27^

It behoves the food safety community, both in the regulatory authorities and in the food industry, to look for a better approach to build confidence and trust with consumers. There are many things to consider including (i) the information to be imparted and (ii) the channels to most affectively access consumers. There is currently a revolution in social media for interacting with the public and for the public to interact with the stakeholders; including online networks, i.e. Facebook, LinkedIn, Wechat, blogs, micro blogs (Twitter and Weibo), video/photo sharing platforms (YouTube, Instagram Flickr, Youku) and social bookmarking sites (Pinterest, Delicious, Reddit), etc.^28–30^

## Risk Communication in crisis situations

There are a litany of cases, from the UK BSE crisis in the 1990s and the 1999 dioxin crisis in Belgium to the more recent 2008 melamine crisis in China and the radiation leakage in Japan in 2011, where governments concealed the full extent of the problem, downplayed the risk and undermined consumer confidence.^3,9,27,31^ Unless the authorities communicate in a more open and transparent way when a crisis occurs trust will be eroded and without a degree of trust the more holistic approach to communication on food- related issues, that we are advocating, will not succeed in delivering consumer confidence and putting risk in perspective.

## “Food Information Communication” to create greater understanding

The word “risk” is naturally negative, and risk communication messages often relate to negative information, which may serve to increase consumers’ anxiety and concern about food. Most of the food agencies focus on risk analysis rather than risk-benefit analysis and often highlight the risks in isolation of the benefits.

Moving from the main focus being on “risk communication” to a broader “food information communication” might afford the opportunity for more positive messages to receive airtime.

The core objectives of food information communication are to establish trust among stakeholders, rebuild consumer confidence, put issues in perspective and inform the public about benefits as well as risks. The big challenge in doing this is not to be considered as patronising to the public. Unless the genuine issues that consumers have concerns about are addressed, it will be very difficult to tackle misperceptions and correct misinformation. Telling people that things are better than they actually are will undermine trust and make the situation worse. Trust has to be earned and cannot be mandated. “Action speak louder than words” and risk management deficiencies need to be addressed if there is to be any hope of building trust and confidence. There are many dimensions to trust and it is not easy to build especially when it has been damaged by industry failures and mismanagement by the authorites.^3,9,11,27,31,32,33^

Food information communication covers a wide range of topics including: (i) The benefits and positive attributes of food and new technologies; (ii) the increased efforts and measures governments are making to improve food monitoring systems and ensure compliance; (iii) efforts by the food industry to improve food quality and safety; (iv) health benefits of food; (v) healthy diets and how to avoid health damage and diseases from unhealthy diets; and (vi) how to properly handle and cook food in commercial kitchens and at home to avoid food-borne diseases. Honesty must remain paramount and the public should also be given information on (vii) potential risks and hazards in food and (viii) receive messages in a timely manner during contaminations incidents, outbreaks of food poisoning, recalls, etc.^34^ Given the relationship between many non-communicable diseases and obesity and inappropriate diets and the prevalence of misleading information on diet, there is a need for a better way to communicate evidence-based information on the nutritional value of foods and new ways to achieve positive change in behaviour.

There is a requirement for improved communication among different government agencies and the industry to avoid unbalanced information, ensure reasonably consistency and prevent contradictions.^35,36^

Novel and innovative scientific developments will only contribute to advances in the food chain if they are acceptable to consumers. There is a challenge to communicate, in a comprehensible fashion, the benefits of new developments to consumers.^37^ Some examples where the perceived risks have completely overshadowed the benefits include (i) irradiation, which is advocated as a useful method to kill spoilage organisms and pathogens in food; (ii) GMOs, which can produce new plant varieties much faster and more accurately than conventional breeding; and (iii) the inclusion of additives and preservatives to enhance the safety of food. Substantial resources are being devoted to food-related research to produce safer and more nutritious food, however before this research can deliver on its potential any new developments have to be acceptable to the public. The stakeholders should actively communicate with the public placing an emphasis on communication during all stages of their research.^38,39^

## The role of social science and communication expertise

The competencies of social scientists are needed to assist in gaining insights into consumer perceptions of risk, and in understanding consumer behaviour and the determinants of trust.

The regulatory agencies and the food industry need to embrace social science to (i) monitor public opinion and concerns, (ii) address these in a timely manner and (iii) enter into dialogue with consumer to ensure their concerns are being addressed satisfactorily. Communication is a two-way process and the many social media channels are now affording the opportunity to proactively engage with the public and receive input from them in real time.

Government officials and other stakeholders along the food chain require training in communication skills, so that they can respond to hot topics of public concern, and communicate about scientific developments vividly in an engaging and reassuring way. We live in an era of sensational media and a few scientists have embraced the new media channels and have become celebrities and Internet stars and are trying to ensure balanced information on the airwaves.^40^ However, a small number of so-called scientists often give opinions in areas outside their expertise and generate misinformation, or pseudoscience, which can be hard to correct.^41^

“No comment”, or weak responses, from the regulators and food scientists often creates a vacuum that is filled with speculation, misinformation and rumours, which can be difficult to rectify once they gain traction. The social media is affording the opportunity for every citizen to become a “journalist and have their opinion, whether it is correct or false, widely disseminated. The regulatory agencies, the academic scientists and the food industry need to embrace the new communication platforms. The key messenger givers, whether organisations or individuals, need to have their entity established and trust built up in peacetime if they are to have credibility in a crisis situation when consumer anxiety is elevated.^42^

Innovative and novel ways of communication are required to make food information more attractive to the public and to interact with the public. One example of a very innovative approach from the world of health care is the “know your lemons” campaign, which uses pictures of lemons to illustrate the different stages of breast cancer. The images which went viral on social media, were launched by a small non-profit organisation, Worldwide Breast Cancer, whose founder tried to find a visually suggestive way to show the physical symptoms of breast cancer without being censored by social media rules or frowned upon.^43^

Innovative education in communities, supermarkets and contact points with the public must be encouraged. Content that allows the audience to participate tends to go viral (K Allocca Ted Talk 2011). A good example is the celebrity Chef Jamie Oliver’s “ad-enough” campaign in the UK, which called for better regulation of junk food ads, aimed at children. The 2018 campaign invited the audience to show their support by posting an image of themselves hiding their eyes on social media with the hash tag #Adenough. It created a social phenomenon that led to the UK Government considering a 9 p.m. watershed for junk food advertising on television, and the Mayor of London promising to ban junk food ads on buses, trains and the underground in London.^44^

Education of children and teenagers should be specially emphasised and an understanding of the food chain should be included on school syllabi. While it is a challenge to make food science exciting, it is not an insurmountable one. The critical period before young people adopt fixed views, and entrenched positions, should be utilised to provide them with balanced information so that they can make informed decisions.

Scientists are great at publishing in “high impact” scientific journals and presenting at technical conferences to their peers. However, it is much more challenging to communicate the excitement and novelty of their scientific breakthroughs to the general public and many scientists do not bother to try. Successful engagement with the public, and the policy makers, using the social media channels may have much higher impact that the impact of the peer-reviewed journals.

## There are many audience requiring different message

The public are but one audience to communicate to and every country’s population is not homogenous with many diverse segments often having different concerns and having different levels of comprehension. Therefore messages have to be customised for the different target audiences.

Similarly, industry stakeholders along the food chain, from farm to fork, range in scale and require appropriately tailored interactions using the most effective channels for the target group.

Increasingly, the authorities are attempting to underpin legislation and policy with robust science but if the formal risk assessments undertaken to assist with this are not communicated clearly, consistently and comprehensibly their usefulness and impact will be greatly diminished.

The front line food safety inspectors in national food control systems operate in the role of the food safety police verifying that food businesses are compliant but could they also play a greater role in the dissemination of accurate information? All elements of the food industry must be aware of the risks if they are to manage them effectively. An understanding of the rationale behind the rules and regulations will make the sectors more conscious of the consequences of non-compliances. Every inspection and interaction with the food industry could be an opportunity to communicate. The front line troops are also an audience for the communications as they are often not up to date with their information and lack knowledge about the latest scientific advances, which can undermine their credibility with food business operators. Competent oversight of the food chain is important as deficiencies undermine public trust in the capabilities of the regulatory agencies. Remembering that communication is a two way process, the national inspectorate can provide insight from the frontline which can inform and improve policy.

Advances in science including whole genome sequencing to track microbes, analytic chemistry that can detect contaminants at lower and lower levels, bioinformatics, big data analytics and real-time surveillance are assisting the regulators and the industry oversee the food chain but the public are blissfully unaware of the new capabilities.

Journalists are the professional communicators and the regulators, and stakeholders along the food chain, need to engage with them, to give them food- related scientific knowledge and help them acquire the competencies to communicate food information accurately.

## The digital Revolution: an opportunity rather than a threat

Holistic “Food Information Communication” is a different approach from conventional risk communication and now there are more communication channels than ever before to both engage with the public and other stakeholders and disseminate information. The communication landscape in all countries is rapidly evolving and the public in all walks of life are increasingly using digital media as their source of information. Scientists’ job promotion and status, in most jurisdictions, depend on the number and quality of their publications in increasingly specialised peer-reviewed scientific journals. Many scientists naively believe that policy makers will search for, find and use, their research to make science-based decisions.^45^ Furthermore, many scientists are reluctant to engage in dialogue in the digital media to correct factual errors, misinformation or oversimplification of issues.^45^

Methodologies for risk assessment and risk management may be easy to transfer between countries however this may not be the case with risk communication where language, culture and politics are all additional influencing factors. Perception of risk, consumers’ concerns and trust vary in different jurisdictions and will require customised solutions.^46,47,48,49^ The evolution of the social media in all geographically areas, albeit at different paces, is providing a new tool in the armoury of those serious about engaging with the public and other stakeholders in the food chain.

The power of the social media is well demonstrated by the on-going impact on vaccination uptake in many jurisdiction based on the findings of a long retracted paper linking autism to measles.^50^ In the food area a short YouTube clip of a celebrity chef, Jamie Oliver, denouncing lean finely textured beef (LFTB) as Pink Slime went viral and heralded the demise of many LFTB plants in the US leading most of the leading food service companies, including McDonalds, to cease using LFTB in their offerings.^51^

Some agencies are seeing the potential in the new media to engage with consumers, and to monitor user generated and shared content and to identify outbreaks of food-borne disease.^52, 53^ However, many food regulatory agencies are slow to aggressively embrace the new channels.

Social media analytics is providing a lot of information about individuals and it is now much easier to segment the population and customise engagement with them. Commercial companies are developing relationship-based interactions with their customers to influence purchasing decisions and perhaps the same techniques could be used to increase understanding on all things relating to food and nutrition and to change public perceptions and behaviour.^54–57^

A further exciting opportunity is to use mobile devices to seek consumer’s views and then, in real time, give them back customised accurate information tailored for their age, gender, educational status, occupation, etc.

There is no limit to the sample size when using mobile networks to access the public and further with big data analytics, it is possible to handle the large data sets and use algorithms linked to personal profiles to give people the exact amount of detail they need and can understand. The commercial marketeers are utilising social media campaigns, and celebrity influencers, to get their messages disseminated and influence consumer behaviour and there are lessons here for the food regulatory agencies.^58,59^

The digital revolution should be seen as an opportunity by the food regulatory authorities in all jurisdictions, rather than a threat.^60,61^ Failure by the authorities to embrace, and use, the new communication platforms and channels will only lead to further undermining of consumer confidence in the food supply as perceptions of the safety of food, of the oversight of the food chain and of the food industry will be based on “alternative facts”, which is the new term for rumours, rather than on accurate information.^62–64^
